# Supplementary material for: EXPERT expands prime editing efficiency and range of large fragment edits
Source: Nat Commun. 2025 Feb 13;16:1592. doi: 10.1038/s41467-025-56734-9 (PMC11822059; doi:10.1038/s41467-025-56734-9)
Supplement: Supplementary file 1 — Supplementary Information [file 41467_2025_56734_MOESM1_ESM.pdf]

Supplementary Figures and Legends

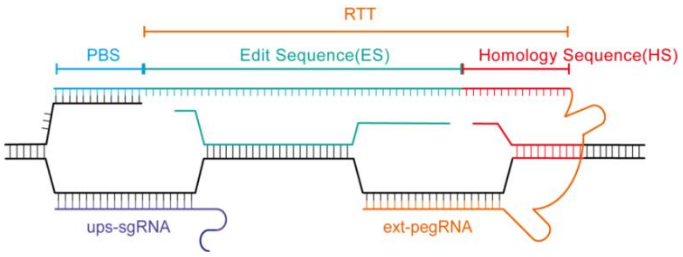

Supplementary Fig. 1 The structural components of ext-pegRNA.

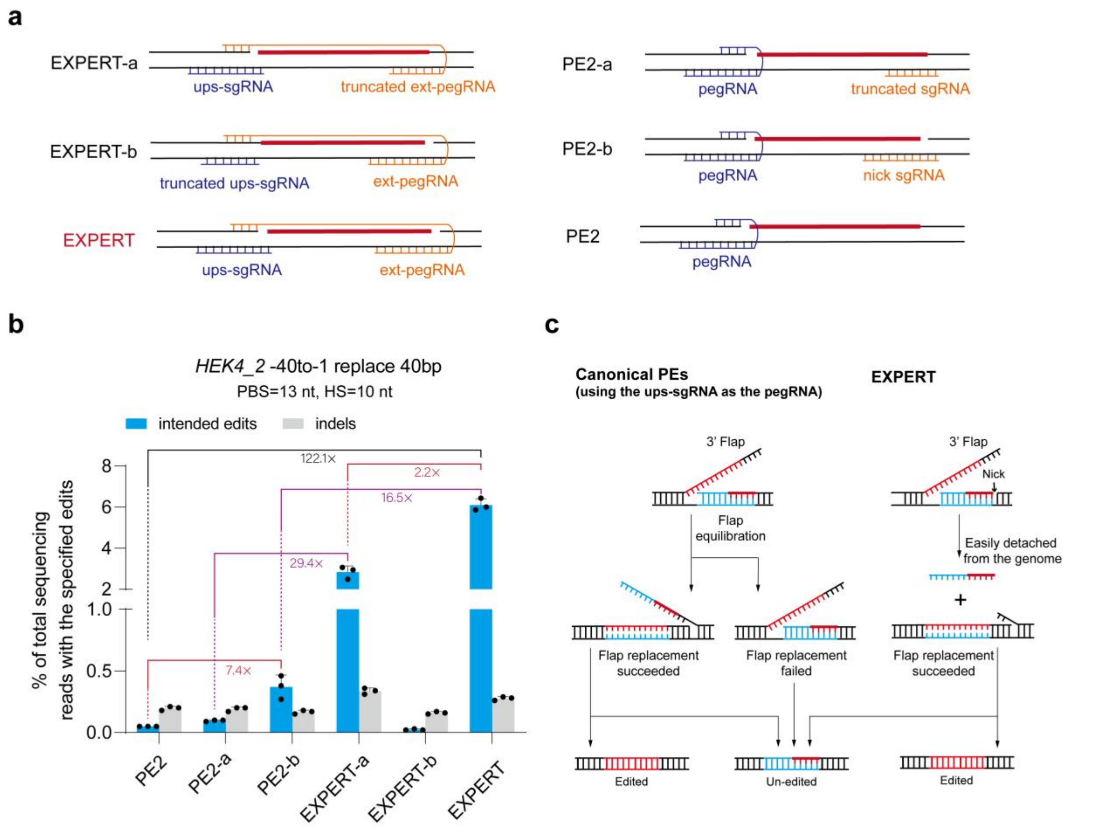

Supplementary Fig. 2 The additional *cis* nick and the upstream binding are necessary for improving the editing efficiency by EXPERT. a Schematic diagram of the composition of several variants (EXPERT-a, EXPERT-b, EXPERT, PE2-a, PE2-b, PE2). These variants have different compositions and modifications. **b** These systems each performed the same replacement of a 40-bp DNA fragment at the *HEK4_2* site. Bars represent the mean of n = 3 independent biological replicates. Data are presented as mean ± s.d. **c** Models for DNA 5' Flap replacement repair of canonical PEs and EXPERT intermediates. In the canonical PEs, if the Flap replacement fails, the non-edited strand will be used as the template to repair the genome; If the Flap replacement succeeds, the editing process will continue. In the EXPERT, an additional nick has been added to the original strand, making it easier for the original strand to detach from the genome, thereby promoting Flap replacement. All sequencing data were collected from transfection-positive cells. Source data are provided as a Source Data file.

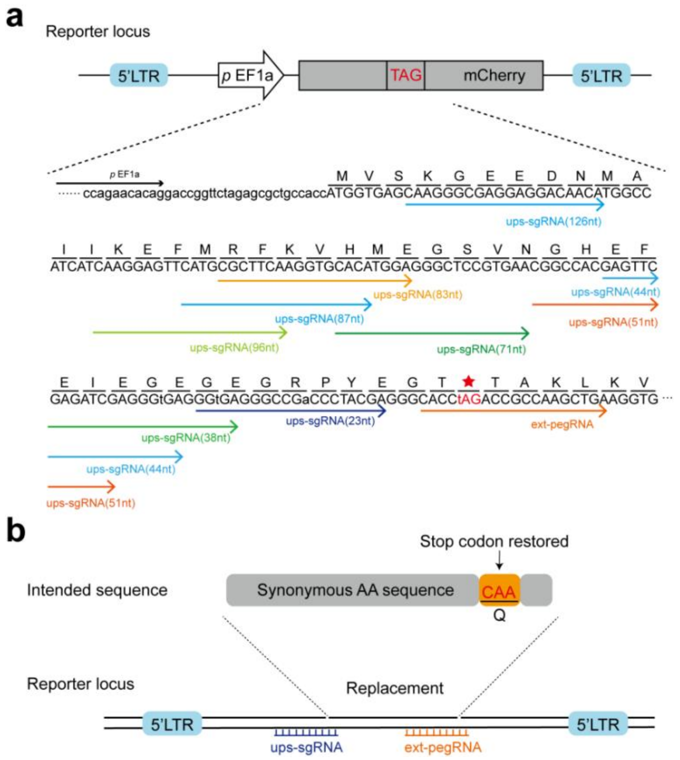

Supplementary Fig. 3 The detailed sequence diagram of the targeting region in 293T-reporter cell line (a) and the pattern diagram of the restoration (b).

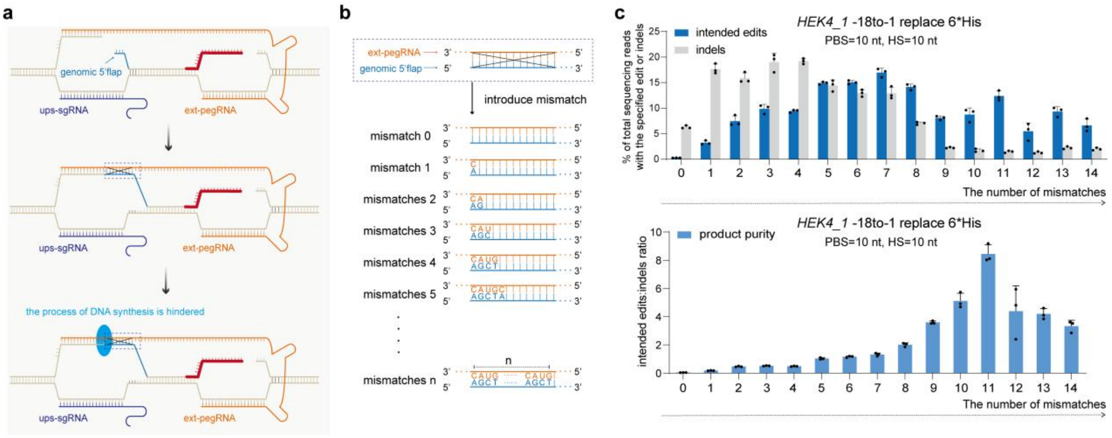

Supplementary Fig. 4 Enhancing EXPERT editing efficiency through the introduction of mismatches on ext-pegRNA. a Diagram of the hybridization of the 5’ Flap of the original DNA strand to complementary ext-pegRNA. The hybridization can also hinder the reverse transcription process of RT enzymes, leading to editing failure. **b** The pattern of introducing mismatch on ext-pegRNA region that hybridizes with the 5’ Flap of the original DNA strand. **c** Frequencies of intended edits and indels introduced by EXPERT under different numbers of mismatches on the ext-pegRNA region that hybridizes with the 5’ Flap (top). The product purity (intended edits: indels ratio) introduced by EXPERT under different numbers of mismatches on the ext-pegRNA region (bottom). Bars represent the mean of n = 3 independent biological replicates. Data are presented as mean ± s.d. All sequencing data were collected from transfection-positive cells. Source data are provided as a Source Data file.

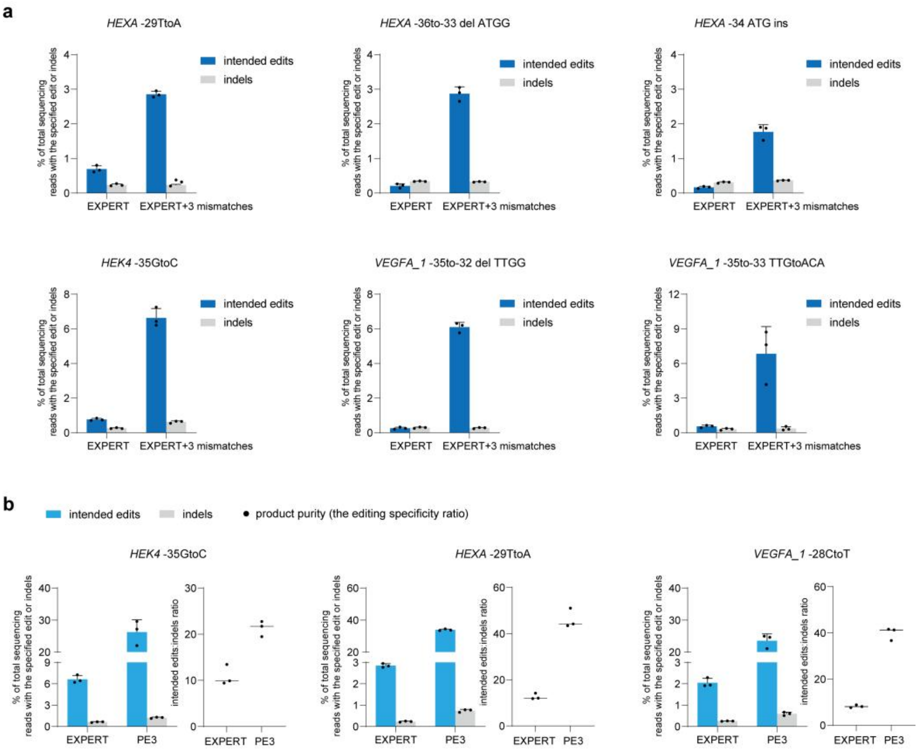

Supplementary Fig. 5 Frequencies of intended small edits and indels introduced by EXPERT and PE3. a Frequencies of intended small edits and indels were performed by EXPERT. Bars represent the mean of n = 3 independent biological replicates. Data are presented as mean ± s.d. **b** Frequencies of intended small edits, indels, and product purity introduced by EXPERT and PE3. Three mismatches were included in EXPERT. Bars represent the mean of n = 3 independent biological replicates. Data are presented as mean ± s.d. All sequencing data were collected from transfection-positive cells. Source data are provided as a Source Data file.

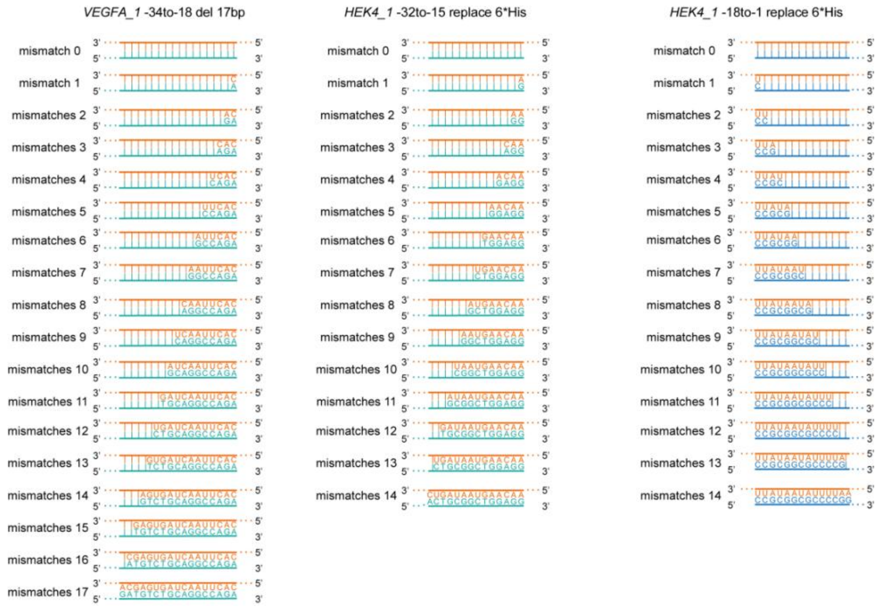

Supplementary Fig. 6 The detailed composition of mismatched nucleotides in Fig. 2e and supplementary Fig. 4c.

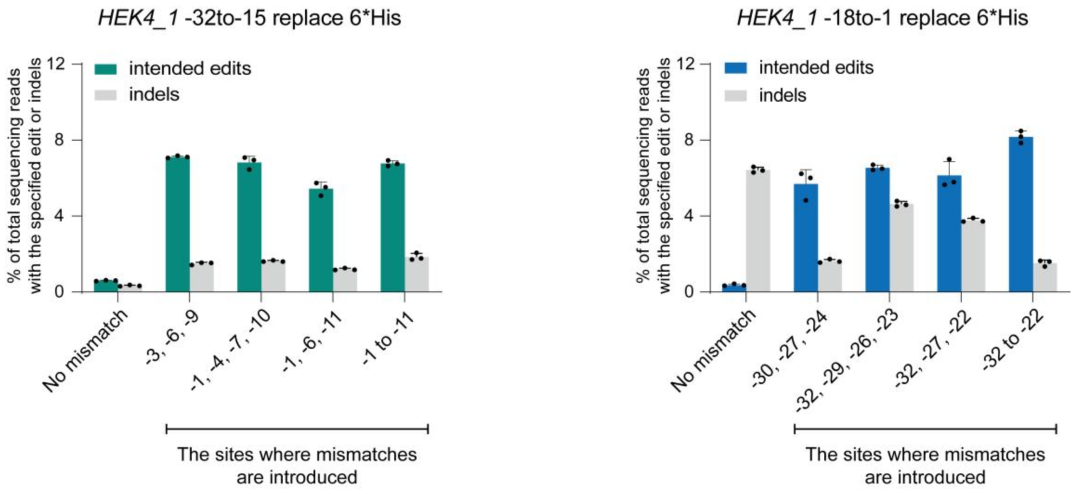

Supplementary Fig. 7 Frequencies of intended edits and indels introduced by EXPERT under different numbers of mismatches on the ext-pegRNA region. Bars represent the mean of n = 3 independent biological replicates. Data are presented as mean ± s.d. All sequencing data were collected from transfection-positive cells. Source data are provided as a Source Data file.

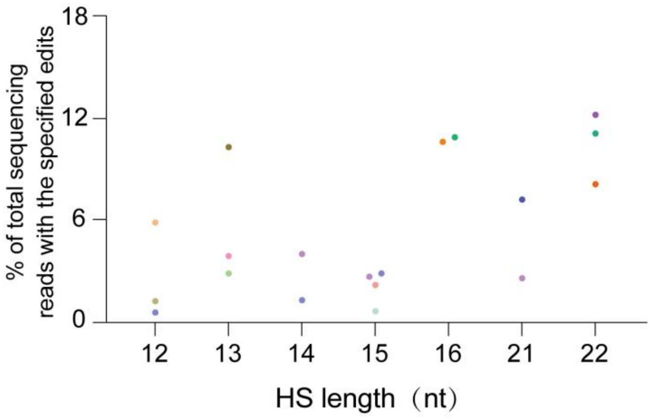

HS length (nt)	≤ 15 nt	≥ 16 nt
Editing efficiency > 6%	1/12	6/7

Supplementary Fig. 8 The investigation of the optimal homology sequence (HS) lengths for EXPERT. The data were collected from the EXPERT data in **Fig. 4a** and **supplementary Fig. 9**. n = 19 editing from independent experiments. When the HS length is shorter than 16 nt, there is only one locus with an editing efficiency greater than 6% (1 out of 12). In contrast, when the HS length is 16 nt or greater, there are six loci with an editing efficiency exceeding 6% (6 out of 7).

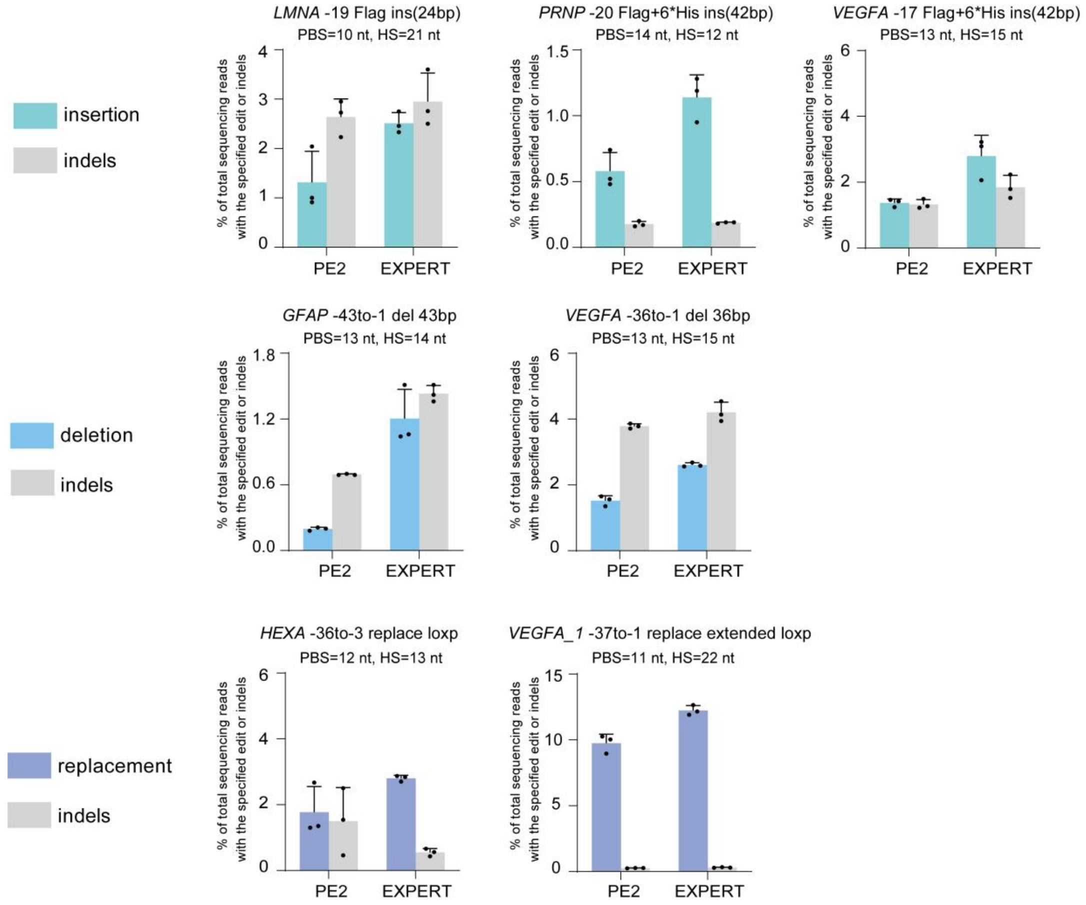

Supplementary Fig. 9 Frequencies of intended edits and indels introduced by PE2 and EXPERT at multiple loci. Bars represent the mean of n = 3 independent biological replicates. Additional mismatches were introduced in the insertion-type edits. Data are presented as mean ± s.d. All sequencing data were collected from transfection-positive cells. Source data are provided as a Source Data file.

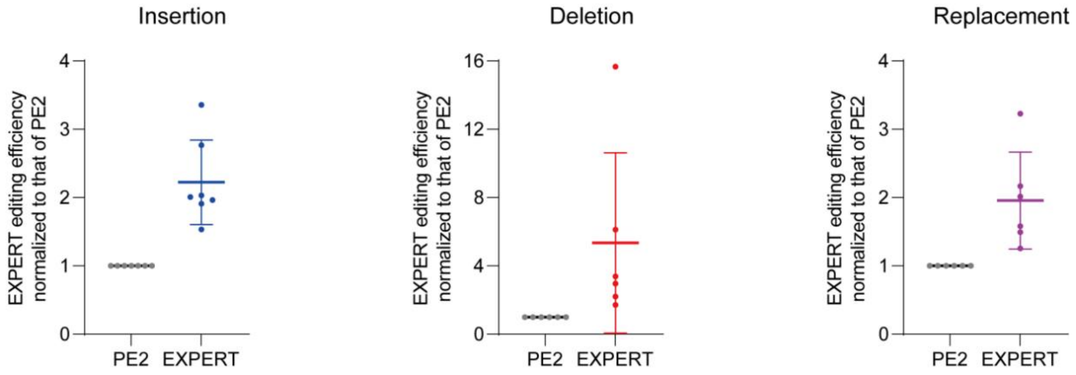

Supplementary Fig. 10 Statistical analysis of normalized editing frequencies of insertions, deletions and replacements , respectively. Setting the frequencies induced by PE2 as 1. n = 6 or 7 editing from independent experiments shown in **Fig. 4a** and **supplementary Fig. 9**, respectively. Data are presented as mean ± s.d.

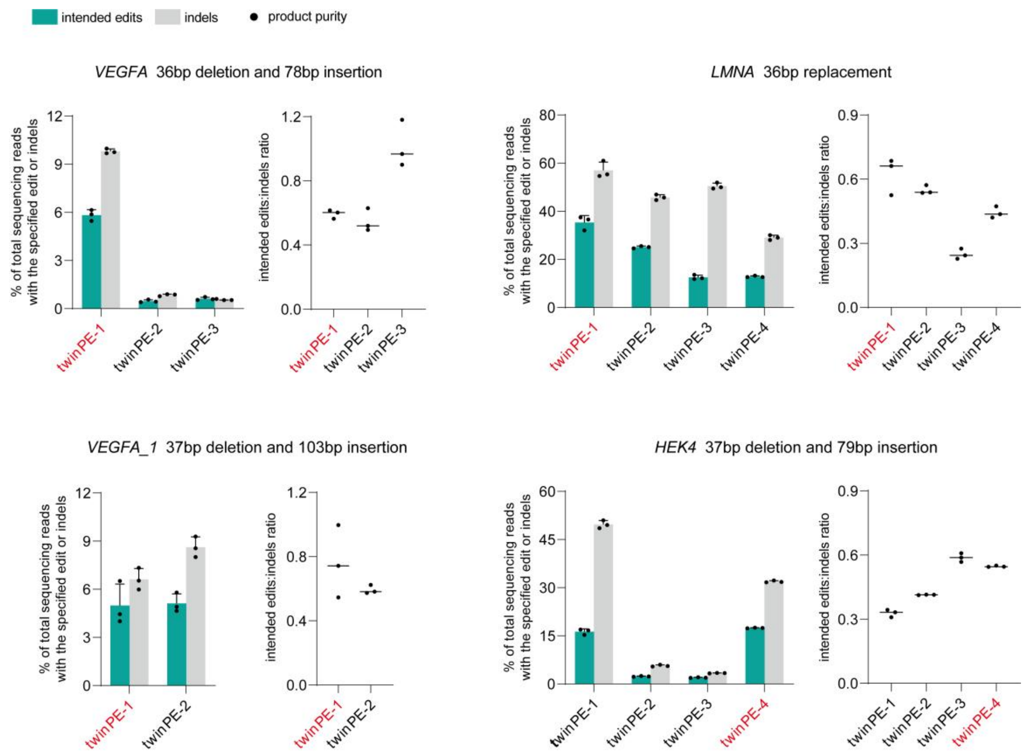

Supplementary Fig. 11 Frequencies of intended edits, indels, and product purity introduced by different twinPE pegRNA pairs at endogenous sites. The red word represents the selected twinPE with the best performance (with precise editing efficiency of at least 2% and the highest product purity). Bars represent the mean of n = 3 independent biological replicates. Data are presented as mean ± s.d. All sequencing data were collected from transfection-positive cells. Source data are provided as a Source Data file.

	EMX1 site					
on-target	intended edits: 9.66% indels: 0.5%					
off-target	ups-sgRNA			ext-pegRNA		
	**1**	**2**	**3**	**1**	**2**	**3**
site1	<0.1	<0.1	<0.1	<0.1	<0.1	<0.1
site2	<0.1	<0.1	<0.1	<0.1	<0.1	<0.1
site3	<0.1	<0.1	<0.1	<0.1	<0.1	<0.1
site4	<0.1	<0.1	<0.1	<0.1	<0.1	<0.1
site5	<0.1	<0.1	<0.1	<0.1	<0.1	<0.1

	VEGFA_1 site					
on-target	intended edits: 11.03% indels: 0.22%					
off-target	ups-sgRNA			ext-pegRNA		
	**1**	**2**	**3**	**1**	**2**	**3**
site1	<0.1	<0.1	<0.1	<0.1	<0.1	<0.1
site2	<0.1	<0.1	<0.1	<0.1	<0.1	<0.1
site3	<0.1	<0.1	<0.1	<0.1	<0.1	<0.1
site4	<0.1	<0.1	<0.1	<0.1	<0.1	<0.1
site5	<0.1	<0.1	<0.1	<0.1	<0.1	<0.1

Supplementary Fig. 12 The gRNA-dependent off-target effects of EXPERT. Potential gRNA-dependent off-target sites were predicted by Cas-OFFinder, allowing up to 5 mismatches. Off-target sites identified by WGS were randomly selected from genome-wide base substitutions.

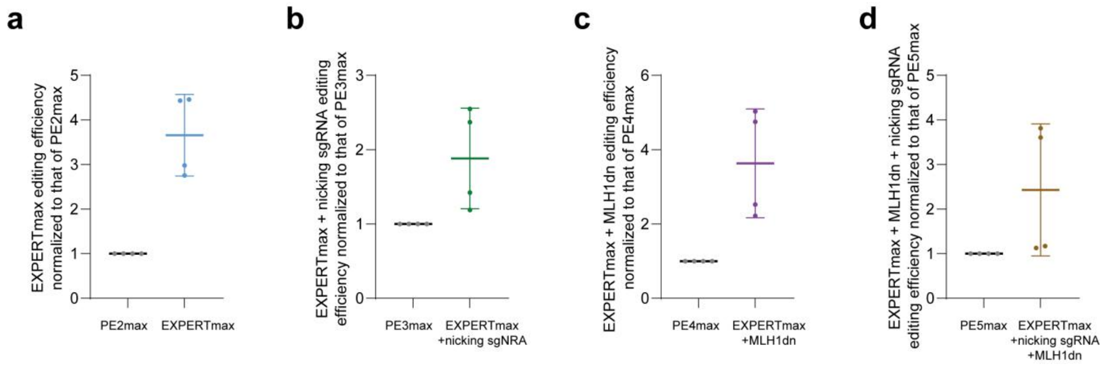

Supplementary Fig. 13 Statistical analysis of normalized editing frequencies of different PEmax and EXPERTmax versions, respectively. a Setting the frequencies induced by PE2max as 1. **b** Setting the frequencies induced by PE3max as 1. **c** Setting the frequencies induced by PE4max as 1. **d** Setting the frequencies induced by PE5max as 1. n = 4 editing from independent experiments shown in **Fig. 5a**, respectively. Data are presented as mean ± s.d.

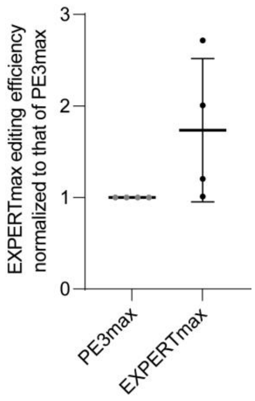

Supplementary Fig. 14 Statistical analysis of normalized editing frequencies of EXPERTmax. Setting the frequencies induced by PE3max as 1. n = 4 editing from independent experiments shown in **Fig. 5a**, respectively. Data are presented as mean ± s.d.

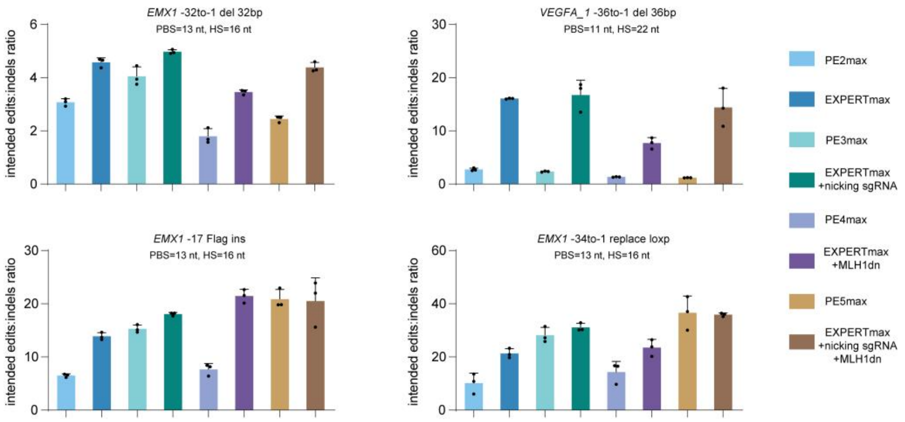

Supplementary Fig. 15 Statistical analysis of product purity of PE2max, PE3max, PE4max, PE5max and their corresponding EXPERTmax systems. Bars represent the mean of n = 3 independent biological replicates. Data are presented as mean ± s.d.

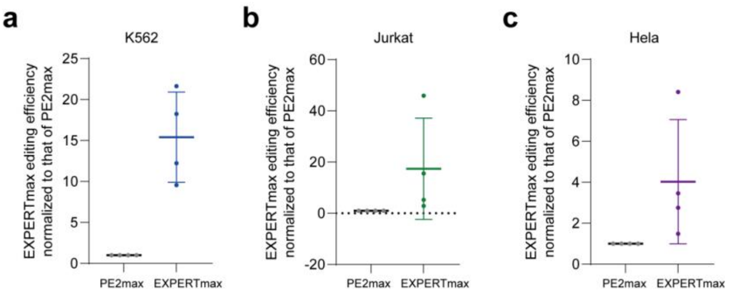

Supplementary Fig. 16 Statistical analysis of normalized editing frequencies of PE2max and EXPERTmax in K562 (a), Jurkat (b) and Hela (c) cells, respectively. a Setting the frequencies induced by PE2max as 1 in K562 cells. n = 4 editing from independent experiments shown in **Fig. 5b**. Data are presented as mean ± s.d. **b** Setting the frequencies induced by PE2max as 1 in Jurkat cells. n = 4 editing from independent experiments shown in **Fig. 5c**. Data are presented as mean ± s.d. **c** Setting the frequencies induced by PE2max as 1 in Hela cells. n = 4 editing from independent experiments shown in **Fig. 5d**. Data are presented as mean ± s.d.

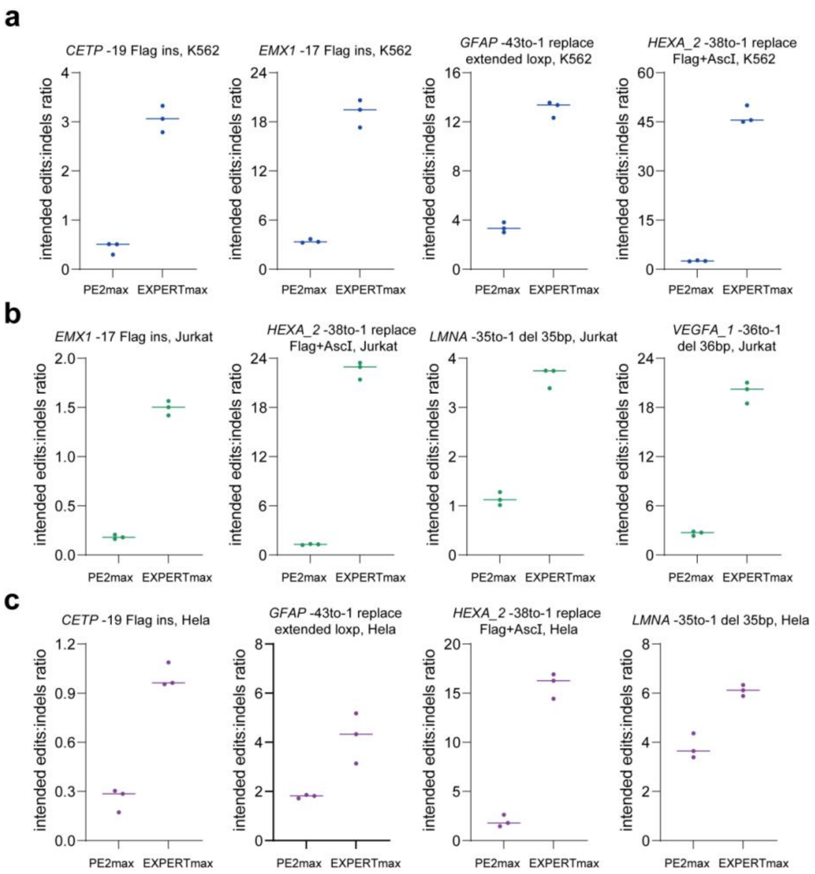

Supplementary Fig. 17 Statistical analysis of product purity of PE2max and EXPERTmax in K562 (a), Jurkat (b) and Hela (c) cells, respectively. a The product purity introduced by PE2max and EXPERTmax in K562 cells. Bars represent the mean of n = 3 independent biological replicates. Data are presented as mean ± s.d. **b** The product purity introduced by PE2max and EXPERTmax in Jurkat cells. Bars represent the mean of n = 3 independent biological replicates. Data are presented as mean ± s.d. **c** The product purity introduced by PE2max and EXPERTmax in Hela cells. Bars represent the mean of n = 3 independent biological replicates. Data are presented as mean ± s.d.

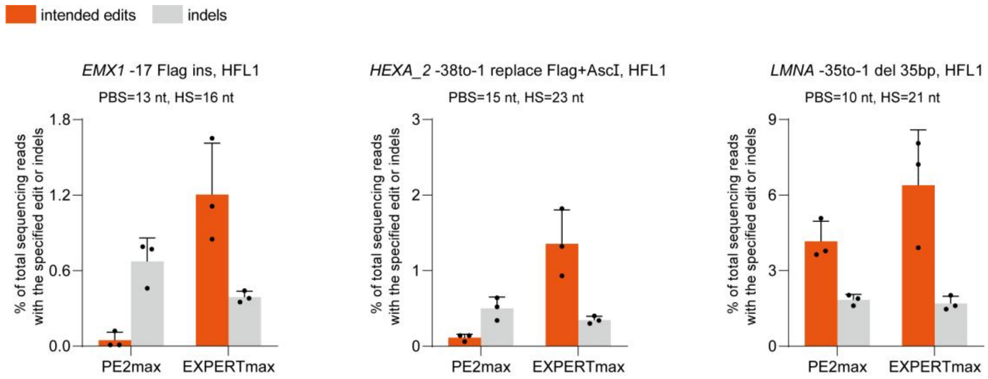

Supplementary Fig. 18 Frequencies of intended edits and indels introduced by PE2max and EXPERTmax at endogenous sites in human fetal lung fibroblast (HFL1) cells. Additional mismatches were introduced in the insertion-type edits. Bars represent the mean of n = 3 independent biological replicates. Data are presented as mean ± s.d. All sequencing data were collected from transfection-positive cells. Source data are provided as a Source Data file.

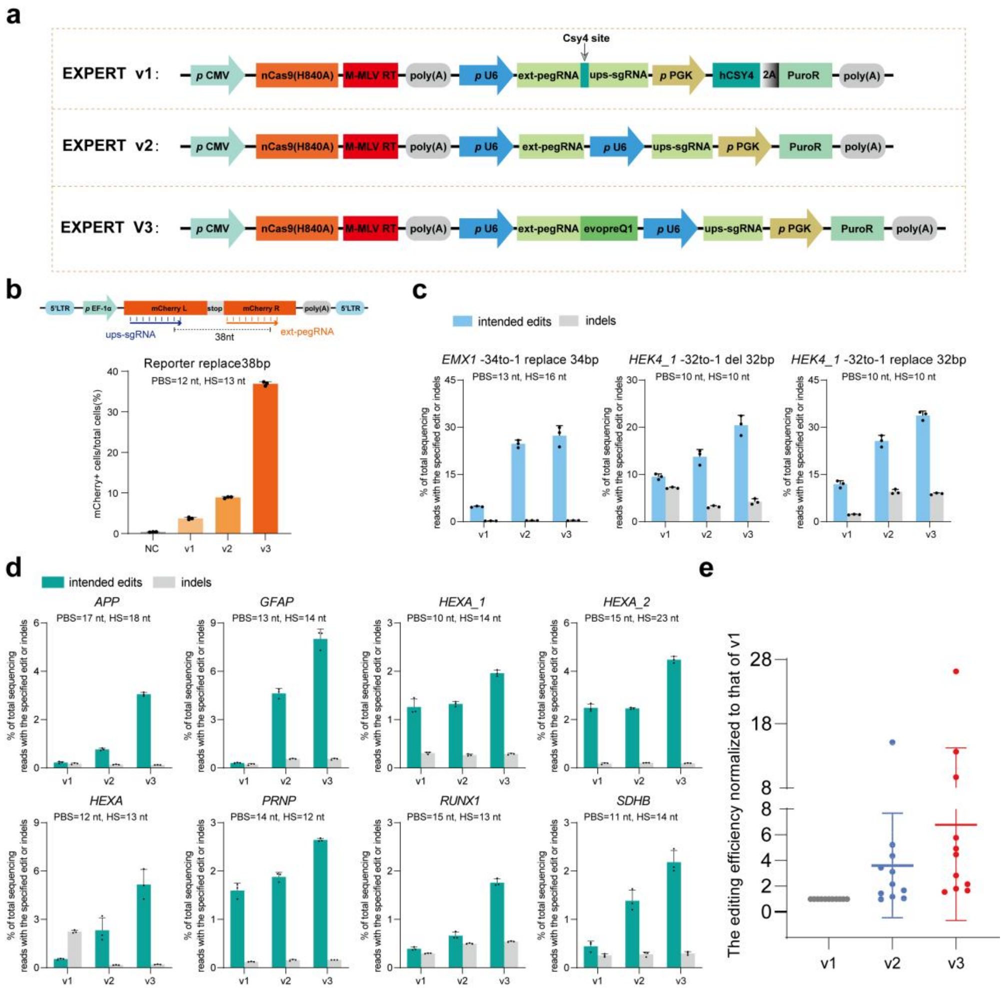

Supplementary Fig. 19 Comparison of three versions of the EXPERT plasmid vector across multiple loci. a Schematic diagram of the structure of the three vector versions of EXPERT. The main difference between these three versions lies in the expression methods of the ext-pegRNA and the ups-sgRNA. EXPERT v1 uses the Csy4 sequence to concatenate ext-pegRNA and ups-sgRNA, while EXPERT v2 uses dual U6 promoters to express them separately. EXPERT v3

adds the evopreQ1 motif on the foundation of EXPERT v2. **b** Diagram of a 38-bp replacement performed by EXPERT in the 293T-reporter cell line containing a stop codon (top). Frequencies of edits introduced by different versions of EXPERT (v1, v2 and v3) were quantified using flow cytometry (bottom). Bars represent the mean of n = 3 independent biological replicates. Data are presented as mean ± s.d. **c** Frequencies of intended edits and indels introduced by different versions of EXPERT (v1, v2 and v3) at *EMX1* and *HEK4_1* sites. Bars represent the mean of n = 3 independent biological replicates. Data are presented as mean ± s.d. **d** Frequencies of replacement edits of the same type but different lengths and indels introduced by different versions of EXPERT (v1, v2 and v3) at eight sites. Bars represent the mean of n = 3 independent biological replicates. Data are presented as mean ± s.d. **e** Statistical analysis of normalized editing frequencies, setting the frequencies induced by EXPERT v1 as 1. n = 11 editing from independent experiments shown in **c** and **d**. Data are presented as mean ± s.d. All sequencing data were collected from transfection-positive cells. Source data are provided as a Source Data file.

Illustration (Supplementary Fig. 19):

In EXPERT, there are two RNA elements (i.e., ups-sgRNA and ext-pegRNA), and the ext-pegRNA is of longer length than the pegRNA employed in the canonical PEs. In the version of EXPERT v1, the ext-pegRNA and ups-sgRNA were concatenated together under one U6 promoter. We tested if separately expressing ups-sgRNA and ext-pegRNA, as shown in EXPERT v2 (Supplementary Fig. 19a), would improve the EXPERT editing efficiency. We further constructed EXPERT v3 based on the v2 version by introducing the evopreQ1 motif to the downstream of the ext-pegRNA (Supplementary Fig. 19a).

We compared the editing efficiencies achieved by EXPERT v1, v2 and v3 in the 293T-reporter cell line. The percentage of mCherry^{+} cells post editing is used as the indicator of successful editing (Supplementary Fig. 3). EXPERT v1 achieved an efficiency of 3.73%. In contrast, EXPERT v2 produced a 2.4-fold increase in editing efficiency (8.9%), and EXPERT v3 displayed a 9.9-fold increase (36.93%) relative to EXPERT v1 (Supplementary Fig. 19b). We then evaluated these three versions by performing different types edits at different endogenous sites: (i) *EMX1* -34to-1 replace 34bp; (ii) *HEK4_1* -32to-1 del 32bp; and (iii) *HEK4_1* -32to-1 replace 32bp in HEK293T cells. Consistently, the v3 version is superior to the v1 and v2 versions (Supplementary Fig. 19c).

To evaluate the performance of EXPERT v1, v2 and v3 at more loci, we compared them in replacement edits ranging from 38-bp to 55-bp in eight endogenous sites. The results showed that both v2 and v3 outperformed version v1 significantly (Supplementary Fig. 19d). Summarizing all three versions of data (Supplementary Fig. 19c and Supplementary Fig. 19d), EXPERT v2 exhibited an overall 3.6-fold improvement over EXPERT v1, and EXPERT v3 an overall 6.8-fold improvement (Supplementary Fig. 19e).

Based on these results, all of three versions are usable, but we recommend the v3 version more.
