# Supplementary material for: EXPERT expands prime editing efficiency and range of large fragment edits
Source: Nat Commun. 2025 Feb 13;16:1592. doi: 10.1038/s41467-025-56734-9 (PMC11822059; doi:10.1038/s41467-025-56734-9)
Supplement: Supplementary file 2 — Description of Additional Supplementary Files [file 41467_2025_56734_MOESM2_ESM.pdf]

**Description of Additional Supplementary Files**

**File Name:** Supplementary Data 1

**Description:** Sequences of ext-pegRNAs, pegRNAs and sgRNAs usedinthis study.

**File Name:** Supplementary Data 2

**Description:** Sequences of all constructs used in this study.

**File Name:** Supplementary Data 3

**Description:** Sequences of PCR primers used in this study.

**File Name:** Supplementary Data 4

**Description:** The information of OT effects in this study.
