# Supplementary material for: EXPERT expands prime editing efficiency and range of large fragment edits
Source: Nat Commun. 2025 Feb 13;16:1592. doi: 10.1038/s41467-025-56734-9 (PMC11822059; doi:10.1038/s41467-025-56734-9)
Supplement: Supplementary file 7 — Reporting Summary [file 41467_2025_56734_MOESM7_ESM.pdf]

nature portfolio

Corresponding author(s): Shuhong Zhao, Jinxue Ruan, Xinyun Li

Last updated by author(s): Jan 16, 2025

Reporting Summary

Nature Portfolio wishes to improve the reproducibility of the work that we publish. This form provides structure for consistency and transparency in reporting. For further information on Nature Portfolio policies, see our Editorial Policies and the Editorial Policy Checklist.

Statistics

For all statistical analyses, confirm that the following items are present in the figure legend, table legend, main text, or Methods section.

n/a	Confirmed	
☐	☒	The exact sample size (*n*) for each experimental group/condition, given as a discrete number and unit of measurement
☐	☒	A statement on whether measurements were taken from distinct samples or whether the same sample was measured repeatedly
☐	☒	The statistical test(s) used AND whether they are one- or two-sided<br>*Only common tests should be described solely by name; describe more complex techniques in the Methods section.*
☐	☒	A description of all covariates tested
☐	☒	A description of any assumptions or corrections, such as tests of normality and adjustment for multiple comparisons
☐	☒	A full description of the statistical parameters including central tendency (e.g. means) or other basic estimates (e.g. regression coefficient) AND variation (e.g. standard deviation) or associated estimates of uncertainty (e.g. confidence intervals)
☐	☒	For null hypothesis testing, the test statistic (e.g. *F*, *t*, *r*) with confidence intervals, effect sizes, degrees of freedom and *P* value noted<br>*Give P values as exact values whenever suitable.*
☒	☐	For Bayesian analysis, information on the choice of priors and Markov chain Monte Carlo settings
☒	☐	For hierarchical and complex designs, identification of the appropriate level for tests and full reporting of outcomes
☒	☐	Estimates of effect sizes (e.g. Cohen's *d*, Pearson's *r*), indicating how they were calculated

Our web collection on statistics for biologists contains articles on many of the points above.

Software and code

Policy information about availability of computer code

Data collection	All sequencing data were acquired using an Illumina NovaSeq 6000.
Data analysis	The obtained amplicon sequencing reads (FASTQ files) were analyzed by CRISPResso2.

For manuscripts utilizing custom algorithms or software that are central to the research but not yet described in published literature, software must be made available to editors and reviewers. We strongly encourage code deposition in a community repository (e.g. GitHub). See the Nature Portfolio guidelines for submitting code & software for further information.

Data

Policy information about availability of data

All manuscripts must include a data availability statement. This statement should provide the following information, where applicable:

- Accession codes, unique identifiers, or web links for publicly available datasets
- A description of any restrictions on data availability
- For clinical datasets or third party data, please ensure that the statement adheres to our policy

All sequencing data have been deposited in the NCBI SRA under project accession number PRJNA1203942.

Human research participants

Policy information about studies involving human research participants and Sex and Gender in Research.

Reporting on sex and gender	*Use the terms sex (biological attribute) and gender (shaped by social and cultural circumstances) carefully in order to avoid confusing both terms. Indicate if findings apply to only one sex or gender; describe whether sex and gender were considered in study design whether sex and/or gender was determined based on self-reporting or assigned and methods used. Provide in the source data disaggregated sex and gender data where this information has been collected, and consent has been obtained for sharing of individual-level data; provide overall numbers in this Reporting Summary. Please state if this information has not been collected. Report sex- and gender-based analyses where performed, justify reasons for lack of sex- and gender-based analysis.*
Population characteristics	*Describe the covariate-relevant population characteristics of the human research participants (e.g. age, genotypic information, past and current diagnosis and treatment categories). If you filled out the behavioural & social sciences study design questions and have nothing to add here, write "See above."*
Recruitment	*Describe how participants were recruited. Outline any potential self-selection bias or other biases that may be present and how these are likely to impact results.*
Ethics oversight	*Identify the organization(s) that approved the study protocol.*

Note that full information on the approval of the study protocol must also be provided in the manuscript.

Field-specific reporting

Please select the one below that is the best fit for your research. If you are not sure, read the appropriate sections before making your selection.

☒ Life sciences ☐ Behavioural & social sciences ☐ Ecological, evolutionary & environmental sciences

For a reference copy of the document with all sections, see nature.com/documents/nr-reporting-summary-flat.pdf

Life sciences study design

All studies must disclose on these points even when the disclosure is negative.

Sample size	Sample sizes were determined based on reported literature for genome editing experiments.
Data exclusions	No data were excluded.
Replication	All experiments were repeated at least three times and attempts at replication were successful.
Randomization	Human cells were grown at identical condition, after seeding cells into 6-wells plates, we randomly selected cell for test group and control group.
Blinding	For molecular and cell experiments, no blinding was applied.

Reporting for specific materials, systems and methods

We require information from authors about some types of materials, experimental systems and methods used in many studies. Here, indicate whether each material, system or method listed is relevant to your study. If you are not sure if a list item applies to your research, read the appropriate section before selecting a response.

Materials & experimental systems

n/a	Involved in the study	
☒	☐	Antibodies
☐	☒	Eukaryotic cell lines
☒	☐	Palaeontology and archaeology
☒	☐	Animals and other organisms
☒	☐	Clinical data
☒	☐	Dual use research of concern

Methods

n/a	Involved in the study	
☒	☐	ChIP-seq
☐	☒	Flow cytometry
☒	☐	MRI-based neuroimaging

March 2021

Eukaryotic cell lines

Policy information about cell lines and Sex and Gender in Research

Cell line source(s)	HEK293T, K562, Jurkat, Hela, N2a, HFL1 and PFF cells
Authentication	HEK293T(ATCC, CRL-11268), K562(ATCC, CRL-3344), Jurkat(ATCC, TIB-152), HeLa(Procell, CL-0101), N2a(Procell, CL-0168), HFL1(Procell, CL-0106) and PFF(isolated by our laboratory from a 35-day-old pig embryo) cells were validated by supplier.
Mycoplasma contamination	All cell lines tested negative for mycoplasma contamination.
Commonly misidentified lines (See ICLAC register)	None misidentified lines were used.

Flow Cytometry

Plots

Confirm that:

☒ The axis labels state the marker and fluorochrome used (e.g. CD4-FITC).

☒ The axis scales are clearly visible. Include numbers along axes only for bottom left plot of group (a 'group' is an analysis of identical markers).

☒ All plots are contour plots with outliers or pseudocolor plots.

☒ A numerical value for number of cells or percentage (with statistics) is provided.

Methodology

Sample preparation	HEK293T, Hela and N2a cells were seeded into 6-well plates and transfected (approximately 80% confluent) with 2.5 μg plasmid using JetPRIME (PolyPlus) according to the manufacturer's instructions. K562, Jurkat, HFL1, and PFF cells were transfected using electroporation instrument (Celetrix). Briefly, a total of 1,000,000 cells were transfected with 2.5 μg plasmid using a 20 μL electroporation cuvette where the cells were shocked with 520 V.<br>K562, Jurkat, and HFL1 cells were enriched with positive cells using flow cytometry sorting. Specifically, At 96 h post-transfection, GFP-positive cells (transfection-positive cells) were FACS-enriched (BD AriaTM III) for subsequent experiments. HEK293T, Hela, N2a, and PFF cells were enriched by adding puromycin (InvivoGen) to the medium at the final concentration of 2 μg/mL, 24 h post-transfection. At 96 h post-transfection, transfection-positive cells were collected and lysed with freshly prepared lysis buffer and incubated at 65 ? for 40 min, followed by incubation at 95 ? for 15 min.
Instrument	BD Aria™ III flow cytometer was used for cell sorting
Software	CytExpert software
Cell population abundance	mCherry activation rates = mCherry positive cell numbers / Total cell numbers.
Gating strategy	Gating strategy for Prime editing analysis, the GFP positive cells were sorted.

☒ Tick this box to confirm that a figure exemplifying the gating strategy is provided in the Supplementary Information.
